# Evaluation of total body water in canine breeds by single-frequency bioelectrical impedance analysis method: specific equations are needed for accuracy

**DOI:** 10.1186/s13104-015-1298-2

**Published:** 2015-08-06

**Authors:** Laurence Yaguiyan-Colliard, Caroline Daumas, Patrick Nguyen, Dominique Grandjean, Philippe Cardot, Nathalie Priymenko, Françoise Roux

**Affiliations:** Breeding and Sport Medicine Unit, Université Paris-Est, École Nationale Vétérinaire d’Alfort, 7 avenue du Général de Gaulle, Maisons-Alfort, 94704 France; Nutrition and Endocrinology Unit, École Nationale Vétérinaire de Nantes (ONIRIS), Atlanpole La Chantrerie, route de Gachet, CS 40706, 44307 Nantes Cedex 3, France; Experimental and Clinical Respiratory Neurophysiology Unit, Faculté de Médecine Pierre and Marie Curie, Université Pierre et Marie Curie, UMRS-1158, 91 Boulevard de l’Hôpital, 75634 Paris Cedex 13, France; École Nationale Vétérinaire UMR1331 Toxalim INRA/INP/UPS-ENVT, BP 87614, 31076 Toulouse Cedex 3, France; Intensive Care Medicine Unit, École Nationale Vétérinaire de Nantes (ONIRIS), Atlanpole La Chantrerie, route de Gachet, CS 40706, 44307 Nantes Cedex 3, France

**Keywords:** Dogs, Bioimpedance analysis, Deuterium dilution, Total body water, Body composition

## Abstract

**Background:**

Equations based on single-frequency bioelectrical impedance analysis at 50 kHz for determination of total body water content (TBW) have been previously validated in healthy non-sedated beagle dogs. We investigated whether these equations are predictive of TBW in various canine breeds by comparing the results of these equations with TBW values evaluated directly by deuterium oxide (D_2_O) dilution.

**Methods:**

Total body water content of 13 healthy adult pet dogs of various breeds was determined directly using D_2_O dilution and indirectly using previous equations based on values obtained with a portable bioelectric impedance device. Paired Student’s *t*-tests were used to compare TBW obtained by single-frequency bioelectrical impedance analysis and D_2_O dilution. A p-value of <0.05 was considered statistically significant for all analyses.

**Results:**

Significant differences were observed between TBW determined by the reference method and the values obtained with both predictive equations.

**Conclusions:**

The proposed equations including single-frequency bioelectrical impedance analysis parameters validated at 50 kHz in healthy adult beagles need to be modified including morphological parameters such as body size and shape in a first approach. As in humans, morphological-specific equations have to be developed and validated.

## Background

Determination of total body water content (TBW) and body composition analysis are essential for meaningful medical follow-up of individuals [1] in physiological (e.g., growth, aging, and sport) as well as pathological (e.g., obesity, dialysis, and disability) states. Therefore, simple and effective techniques for evaluation of body composition are highly desirable.

Bioelectrical impedance analysis (BIA) is often presented as an alternative to dilution techniques and to dual X-ray absorptiometry for the evaluation of TBW and body composition. It is a quick non-invasive technique that requires inexpensive equipment and a low-intensity electrical current, which is painless and undetectable by the subject. BIA has been validated and routinely used in healthy as well as sick people [2–4]. This technique has also been validated with various types of equipment in several species including dogs [5–8], which has made it possible to derive linear regression equations for TBW evaluation. Nevertheless, development of specific equations in humans based on sex, age, ethnic group, and physical activity has been necessary [9]. No similar data for dogs have been published.

Because BIA has potential as an easy technique for assessment of body composition in veterinary practice, we aimed to verify whether the protocol and equations validated in adult non-anesthetized beagle dogs for single-frequency (SF-BIA) at 50 kHz [8] were applicable for the prediction of TBW in non-anesthetized dogs of various breeds.

## Methods

### Subjects

Thirteen adult dogs of various breeds (3 middle-sized crossbred dogs, 2 Australian shepherd dogs, 2 Siberian huskies, 2 golden retrievers, 1 miniature schnauzer, 1 Yorkshire terrier, 1 Belgian shepherd dog, and 1 English bulldog) owned and voluntarily provided by veterinary students were used in the study. A written informed consent was obtained from each owner. The dogs were 4.3 ± 2.9 years (mean ± SD) in age and weighed 20.97 ± 8.09 kg (mean ± SD). The mean body condition score (9-point scale BCS) was 5.3/9 with a range of 5/9 to 7/9. The experimental protocols adhered to European Union ethical guidelines and were approved by the Institute of Animal Clinical Research of Alfort.

### Deuterium dilution technique

Food and water were restricted from 2 h before to 4 h after injection of the tracer. TBW was measured using the isotopic dilution of deuterium (D_2_O). On the day of the measurement, 12-h food-deprived dogs were injected subcutaneously in the dorsal cervical region with D_2_O [99.9% H/H^2^; 500 mg/kg body weight (W); Eurisotop, Gif-sur-Yvette, France] prepared with physiological saline solution (9 g NaCl/L). Syringes were weighed before and after administration of D_2_O to determine the exact dose administered, with an accuracy of 0.1 g. Blood samples (5 mL) were collected in EDTA tubes from the jugular vein before and 4 h after injection of the tracer [10]. Plasma was separated by centrifugation at 2,000×*g* for 10 min, and stored at −20°C in sealed vials until analysis. Plasma D_2_O was assayed in duplicate using Fourier-transform infrared spectroscopy, as validated and described previously by Ferrier et al. [11].

### Blood testing

Blood samples (2 mL) were collected in heparin tubes from the jugular vein before injection of the tracer for determination of hematocrit and blood glucose, total protein, albumin, sodium, potassium, and chloride levels.

### Body measurements

Following injection of the tracer, the animals were weighed and measured. BCS for each dog was evaluated by the same trained veterinarian. Body length (L; from the external occipital protuberance to the base of the tail) was determined using a flexible tape. Thorax (rib-cage) circumference (RC; at the xyphoid process) and abdominal circumference (AC; at the umbilicus) were measured using a self-rewind tape (Holtex+, Aix-en-Provence, France) when the animal was in full expiration. Scapula height (H) was measured from the ground to the dorsal border of the scapula with a portable stadiometer (Tanita France SA, Neuilly-sur-Seine, France). All measurements were recorded in centimeters (±1/10) and weights in kilograms (±1/100).

### SF-BIA

The animal was placed in a non-electrically conductive harness and then connected to the equipment through four electrodes clamped on the right fore- and hindlimb as previously described by Yaguiyan-Colliard et al. [8]. The dog did not have to be restrained by an operator. The structure was placed on an electrically insulating mat so the animal did not contact any electrically conductive object.

Each animal was subjected to three sequential measurements of impedance with the bioelectrical impedance device (Z-Métrix, BioparHom, Bourget du Lac, France) with a 77 µA current and 50 kHz frequency. The dogs were restrained in the harness for about 5 min for the procedure. Sequential measurements also enabled investigation of the repeatability of the measurements. Data were transmitted directly from the analyzer to a computer and stored in a spreadsheet format file (Microsoft Office Excel, Microsoft France, Issy-les-Moulineaux, France).

### Statistical analysis

Repeatability of measurements refers to the variation in repeat measurements made on the same subject under identical conditions. Variability in measurements made on the same subject can then be ascribed only to errors related to the measurement process itself. The repeatability of R and X measurements in each of the 13 dogs in the study was evaluated by the coefficient of variation (CV) as 100 times the ratio between the standard deviation (SD) and the mean of the R and X measurements, respectively.

The two predictive equations for TBW (1) and (2) were previously obtained by linear regression and validated in adult beagle dogs [8].

1 $${\text{TBW}}_{1} = - 0.019\left( {{\text{L}}/{\text{R}}} \right) + - 0.199\left( {{\text{RC}} + {\text{AC}}} \right) + 0.996{\text{ ~W}} + 0.081{\text{H}} + 12.31$$

2 $${\text{TBW}}_{2} = 0.048\left( {{\text{L}}/{\text{R}}} \right) + - 0.144\left( {{\text{RC}} + {\text{AC}}} \right) + 0.777{\text{ }}~{\text{W}} + 0.066{\text{H}} + 0.031{\text{X}} + 7.47$$

The TBW values computed using impedance measurements (TBW_i_) with the two equations and the TBW value measured directly by isotopic dilution (TBW_d_) were calculated. We used paired Student’s *t*-tests to compare TBW obtained by SF-BIA and dilution (reference method). A p-value of <0.05 was considered statistically significant for all analyses.

## Results

The owners’ dogs remained quiet in the restraining device without operator interference and tolerated the electrodes for the duration of the measurements.

Blood test results were found to be within normal ranges in all the dogs (data not shown).

The physical characteristics of the dogs and values obtained (Table 1) were used to calculate the TBW by using the previously validated Eqs. (1) and (2).

**Table 1:** Parameters of the 13 dogs

Dogs	Breed	Sex	Age (years)	W (kg)	L (cm)	RC (cm)	AC (cm)	H (cm)
1	Mixed breed	M	7.00	32.50	90	76.6	55	64.4
2	Australian shepherd	MN	3.75	18.95	69.2	63.3	50.3	56.6
3	Australian shepherd	M	3.00	28.30	73.8	70.8	55.8	52.3
4	Siberian husky	FN	1.00	17.10	70.3	62.6	46.3	52.5
5	Miniature schnauzer	M	1.30	6.95	40.2	45.5	36.4	36.2
6	Mixed breed	MN	2.00	21.50	64.8	66.2	53.5	50.7
7	Golden retriever	M	6.50	27.80	76.1	73.3	56.6	54.7
8	Yorkshire terrier	M	10.90	5.15	43.7	40.2	34.1	26.1
9	Golden retriever	MN	4.70	27.30	79.6	71.8	58	58.8
10	Siberian husky	M	2.70	19.50	80.3	62.2	45.9	58.6
11	Mixed breed	FN	4.10	17.65	61.7	62	49.3	48.7
12	Belgian shepherd dog	FN	2.20	25.60	75.6	70.5	51.9	60.3
13	English bulldog	FN	7.20	24.25	58	65.9	56.4	35.9
		Mean	4.33	20.97				
		SD	2.86	8.09				

*M* male, *MN* neutered male, *F* female, *FN* neutered female.

The median CV was 2.90% (range 0.27–9.54%) for R and 2.38% (range 0.05–10.56%) for X (Table 2).

**Table 2:** Bioimpedance parameters and TBW values obtained in the study dogs with the two methods

Dogs	R (Ω)	SD_R_ (Ω)	CV_R_	X (Ω)	SD_X_ (Ω)	CV_X_	TBW_d_ (L)	TBW_1_ (L)	Diff_1_ (L)	TBW_2_ (L)	Diff_2_ (L)
1	97.87	0.30	0.30	22.70	0.24	1.08	19.46	22.14	2.68	22.65	3.19
2	188.42	1.48	0.77	37.65	0.28	0.73	11.41	12.68	1.27	11.91	0.50
3	127.78	4.00	3.14	32.95	3.48	10.56	12.60	18.73	6.13	17.70	5.10
4	164.83	12.46	7.56	59.52	0.03	0.05	6.35	11.35	5.00	11.77	5.42
5	108.70	0.29	0.27	27.75	0.22	0.79	4.45	5.58	1.13	4.99	0.53
6	84.68	0.99	1.17	21.12	0.17	0.80	13.11	13.07	−0.04	13.27	0.16
7	23.43	2.24	9.54	298.51	5.52	1.85	15.90	13.88	−2.02	35.04	19.14
8	135.00	2.75	2.04	27.86	1.13	4.05	3.01	4.50	1.49	3.99	0.98
9	100.13	1.64	1.64	36.04	0.56	1.56	15.78	17.23	1.45	17.98	2.20
10	148.38	1.21	0.81	33.49	0.29	0.86	12.10	14.14	2.04	14.00	1.89
11	142.84	4.44	3.11	33.06	0.53	1.60	8.51	11.18	2.67	10.63	2.12
12	117.06	5.83	4.98	27.30	0.72	2.64	15.01	17.41	2.39	16.86	1.84
13	104.77	2.44	2.33	17.77	0.78	7.38	13.15	14.42	1.27	13.11	−0.04
Mean ± SD			2.90			2.38			1.96 ± 2.04		3.31 ± 5.06

Mean and SD of R and X as well as TBW determined directly by D_2_O dilution (TBW_d_) and indirectly with Eqs. (1) (TBW_1_) and (2) (TBW_2_) with values obtained using a portable bioelectric impedance device [resistance (R) and reactance (X)] and morphological parameters. Diff_1_ and Diff_2_: differences between the TBW_d_ value and the TBW values computed with Eqs. (1) and (2), respectively.

Applying the two equations to the group of dogs demonstrated an overestimation of TBW compared with data obtained by isotopic dilution. The mean difference was 1.96 ± 2.04 L (Diff_1_) for the first equation and 3.31 ± 5.06 L (Diff_2_) for the second equation (Table 2). This corresponds to differences of 14.4 and 22.2% between computed TBW_1_ and TBW_2_, respectively, and TBW_d_.

Student’s *t*-tests were applied to the values obtained by the two equations. The values obtained by dilution were significantly different from those obtained using SF-BIA (p_1_ = 0.0046 for the first equation and p_2_ = 0.036 for the second equation) (Table 2).

## Discussion

A simple system was developed in the previous study for restraint for SF-BIA measurements in beagles [8]. This system efficiently maintained the study dogs of various breeds standing on four legs in a physiological position, without any discomfort, for the few seconds the measurements required. This is an important point because the envisaged future benefit of this study is the use of BIA in field conditions.

In this study, SF-BIA at 50 kHz was applied in non-laboratory healthy adult dogs of various breeds. The BIA system could be applied in non-sedated dogs without generating unnecessary stress in the animals. The TBW values obtained with predictive equations based on BIA and morphological parameters were significantly different from those obtained with D_2_O dilution.

In our previous study on 26 beagle dogs with the same protocol and device [8], the mean CVs were slightly lower than those of the present study (1.6 vs. 2.9% for R and 2.2 vs. 2.38% for X). However coefficients of variation for R and X found in this study were low, which illustrates that the precision of the BIA method was good with the study dogs in spite of the variety of breeds and coats, and a lower compliancy than laboratory dogs.

Bioelectrical impedance analysis may be affected by many variables such as hydration status, consumption of food and water, skin and air temperature, recent physical activity, conductance of the examination table, and posture, in addition to electrode positioning and instrumentation [12–14]. This study was designed to standardize and control as many of these variables as possible. No alterations were found in blood parameters in any of the dogs that could have altered the conduction of electric current in the body (data not shown).

However, the diversity of shapes and coats of canine breeds as well as the body condition of the dogs might explain the inaccuracy of predictive equations for TBW validated in beagle dogs observed in this study. One dog, a Braque Français cross (dog no. 6; Tables 1, 2), showed similar results for TBW between the two methods. This dog, although larger, had the same shape and coat as a beagle dog.

## Conclusions

Body shape and condition as overweight impacted the results, albeit the dogs used in this study had an average normal body condition.

This study showed that in dogs, as in humans, it is necessary to define predictive equations for TBW based on BIA parameters according to various morphological parameters such as body size and body condition. Further study is needed to determine coefficients for the equations in dogs according to variables such as breed, sex, condition, activity level, and age.

## Abbreviations

AC: abdominal circumference
BCS: body condition score
BIA: bioimpedance analysis
CV: coefficient of variation
D_2_O: deuterium
H: scapula height
L: body length
R: resistance
RC: rib-cage circumference
SD: standard deviation
SF-BIA: single-frequency bioelectrical impedance analysis
TBW: total body water
W: body weight
X: reactance
Z: impedance

## References

[1] Thibault R, Pichard C (2012). The evaluation of body composition: a useful tool for clinical practice. Ann Nutr Metab.

[2] Jaffrin MY (2009). Body composition determination by bioimpedance: an update. Curr Opin Clin Nutr Metab Care.

[3] Casey AF (2013). Measuring body composition in individuals with intellectual disability. J Obes.

[4] Böhm A, Heitmann BL (2013). The use of bioelectrical impedance analysis for body composition in epidemiological studies. Eur J Clin Nutr.

[5] Sheltinga MR, Helton WS, Rounds J, Jacobs DO, Wilmore DW (1991). Impedance electrodes positioned on proximal portions of limbs quantify fluid compartments in dogs. J Appl Physiol.

[6] Burkholder WJ (1994) Body composition of dogs determined by carcass composition analysis, deuterium oxide dilution, subjective and objective morphometry, and bioelectrical impedance. Ph.D. thesis, Virginia Polytechnic Institute and State University, Blacksburg, p 357

[7] Free Patents Online Web Site (2014) Pet body fat measuring tool: United States patent application 20090076408. http://www.freepatentsonline.com/20090076408.pdf. Accessed 2 Dec 2014

[8] Yaguiyan-Colliard L, Daumas C, Bousbiat S, Jaffrin M, Cardot P, Grandjean D (2015). Indirect assessment of total body water in adult beagle dogs by single-frequency bioelectrical impedance analysis (SF-BIA). Am J Vet Res.

[9] Ward LC (2012). Segmental bioelectrical impedance analysis: an update. Curr Opin Clin Nutr Metab Care.

[10] Jeusette I, Daminet S, Nguyen P, Shibata H, Saito M, Honjoh T (2006). Effect of ovariectomy and ad libitum feeding on body composition, thyroid status, ghrelin and leptin plasma concentrations in female dogs. J Anim Physiol Anim Nutr.

[11] Ferrier L, Robert P, Dumon H, Martin L, Nguyen P (2002). Evaluation of body composition in dogs by isotopic dilution using a low-cost technique, Fourier-transform infrared spectroscopy. J Nutr.

[12] Gudivaka R, Schoeller DA, Kushner RF, Bolt MJG (1999). Single- and multifrequency models for bioelectrical impedance analysis of body water compartments. J Appl Physiol.

[13] O’Brien C, Baker-Fulco CJ, Young AJ, Sawka MN (1999). Bioimpedance assessment of hypohydration. Med Sci Sports Exerc.

[14] Scharfetter H, Monif M, László Z, Lambauer T, Hutten H, Hinghofer-Szalkay H (1997). Effect of postural changes on the reliability of volume estimations from bioimpedance spectroscopy data. Kidney Int.

